# Brain abscess caused by Actinomyces turicensis in a non-immunocompromised adult patient: a case report and systematic review of the literature

**DOI:** 10.1186/s12879-024-08995-w

**Published:** 2024-01-20

**Authors:** Alessandra Imeneo, Lorenzo Vittorio Rindi, Andrea Di Lorenzo, Rosario Alessandro Cavasio, Pietro Vitale, Ilaria Spalliera, Mario Dauri, Daniele Guerino Biasucci, Ilaria Giuliano, Cartesio D’Agostini, Silvia Minelli, Maria Cristina Bossa, Anna Altieri, Massimo Andreoni, Vincenzo Malagnino, Marco Iannetta, Loredana Sarmati

**Affiliations:** 1 Infectious Diseases, Department of Systems Medicine, Tor Vergata University, Rome, Italy; 2 Infectious Disease Clinic, Policlinico Tor Vergata, Rome, Italy; 3 Department of Anaesthesiology, Emergency and Intensive Care Medicine, Policlinico Tor Vergata, Rome, Italy; 4 Department of Clinical Sciences and Translational Medicine, Tor Vergata University, Rome, Italy; 5 Emergency and Reception Department, Anesthesia and Resuscitation Unit, Policlinico Tor Vergata, Rome, Italy; 6 Laboratory of Clinical Microbiology, Policlinico Tor Vergata, Rome, Italy

**Keywords:** *Actinomyces turicensis*, *Schaalia*, Actinomycosis, Systematic review, Case report

## Abstract

**Background:**

*Actinomyces turicensis* is rarely responsible of clinically relevant infections in human. Infection is often misdiagnosed as malignancy, tuberculosis, or nocardiosis, therefore delaying the correct identification and treatment. Here we report a case of a 55-year-old immunocompetent adult with brain abscess caused by *A. turicensis*. A systematic review of *A. turicensis* infections was performed.

**Methods:**

A systematic review of the literature was performed according to the Preferred Reporting Items for Systematic Reviews and Meta-Analyses (PRISMA) guidelines. The databases MEDLINE, Embase, Web of Science, CINAHL, Clinicaltrials.gov and Canadian Agency for Drugs and Technology in Health (CADTH) were searched for all relevant literature.

**Results:**

Search identified 47 eligible records, for a total of 67 patients. *A. turicensis* infection was most frequently reported in the anogenital area (*n* = 21), causing acute bacterial skin and skin structure infections (ABSSSI) including Fournier’s gangrene (*n* = 12), pulmonary infections (*n* = 8), gynecological infections (*n* = 6), cervicofacial district infections (*n* = 5), intrabdominal or breast infections (*n* = 8), urinary tract infections (*n* = 3), vertebral column infections (*n* = 2) central nervous system infections (*n* = 2), endocarditis (*n* = 1). Infections were mostly presenting as abscesses (*n* = 36), with or without concomitant bacteremia (*n* = 7). Fever and local signs of inflammation were present in over 60% of the cases. Treatment usually involved surgical drainage followed by antibiotic therapy (*n* = 51). Antimicrobial treatments most frequently included amoxicillin (+clavulanate), ampicillin/sulbactam, metronidazole or cephalosporins. Eighty-nine percent of the patients underwent a full recovery. Two fatal cases were reported.

**Conclusions:**

To the best of our knowledge, we hereby present the first case of a brain abscess caused by *A. turicensis* and *P. mirabilis*. Brain involvement by *A. turicensis* is rare and may result from hematogenous spread or by dissemination of a contiguous infection. The infection might be difficult to diagnose and therefore treatment may be delayed. Nevertheless, the pathogen is often readily treatable. Diagnosis of actinomycosis is challenging and requires prompt microbiological identification. Surgical excision and drainage and antibiotic treatment usually allow for full recovery.

**Supplementary Information:**

The online version contains supplementary material available at 10.1186/s12879-024-08995-w.

## Background

*Actinomyces are* filamentous Gram-positive anaerobic bacteria [1], generally found as commensals of the oropharynx and gastrointestinal or urogenital tracts [2]. Actinomycosis is a non-opportunistic and generally polymicrobial progressive granulomatous disease, characterized by subacute or chronic abscess formation, frequently misdiagnosed as malignancy, tuberculosis, or nocardiosis [1, 3]. It is characterized by tiny yellow clumps called *sulfur granules*, constituted by a biofilm of bacteria. These, together with necrosis and filamentous Gram-positive fungal-like bacteria, are the typical microscopic findings [3].

Actinomycosis generally involves the cervicofacial region (50%), the thoraco-pulmonary (30%) or the abdominopelvic tract (20%) [1]. The infection is acquired by minor trauma or aspiration rather than via hematogenous spread [4]. *Actinomyces israelii* is the most common species in human infections and in most clinical forms of actinomycosis, while *A. turicensis* is rarely responsible for clinically relevant infections in humans [3, 4].

The disease is generally readily treatable but often misdiagnosed [2]. The microbiological identification of the pathogen is mandatory, especially since the infection is often polymicrobial. In addition to culture, which takes at least 5 days and up to 15–20 days and could frequently result sterile, genotypic methods, such as comparative 16S ribosomal RNA (rRNA) gene sequencing and matrix-assisted laser desorption ionization time-of-flight (MALDI-TOF), are quicker and more accurate tools for *Actinomyces* identification. *Actinomyces* generally retain sensitivity to a wide spectrum of antimicrobials, including β-lactams, clarithromycin, erythromycin, doxycycline, and clindamycin. Long-term treatments are generally required, in addition to surgical debridement.

We report the case of a 55-year-old man with polymicrobial brain abscesses involving *Actinomyces turicensis*; to the best of our knowledge this is the first case in an adult patient with a history of previous alcohol abuse but no other reasons for immunosuppression. We also performed a systematic review of the literature, to summarize cases of infections due to *A. turicensis.* Because of the paucity of reports, we believe this work might be of interest to Infectious Diseases and Internal Medicine practitioners, to better understand the clinical presentations, diagnostic approach, and current treatment strategies of actinomycosis due to *A. turicensis*.

## Case report

**Fig. 1:**
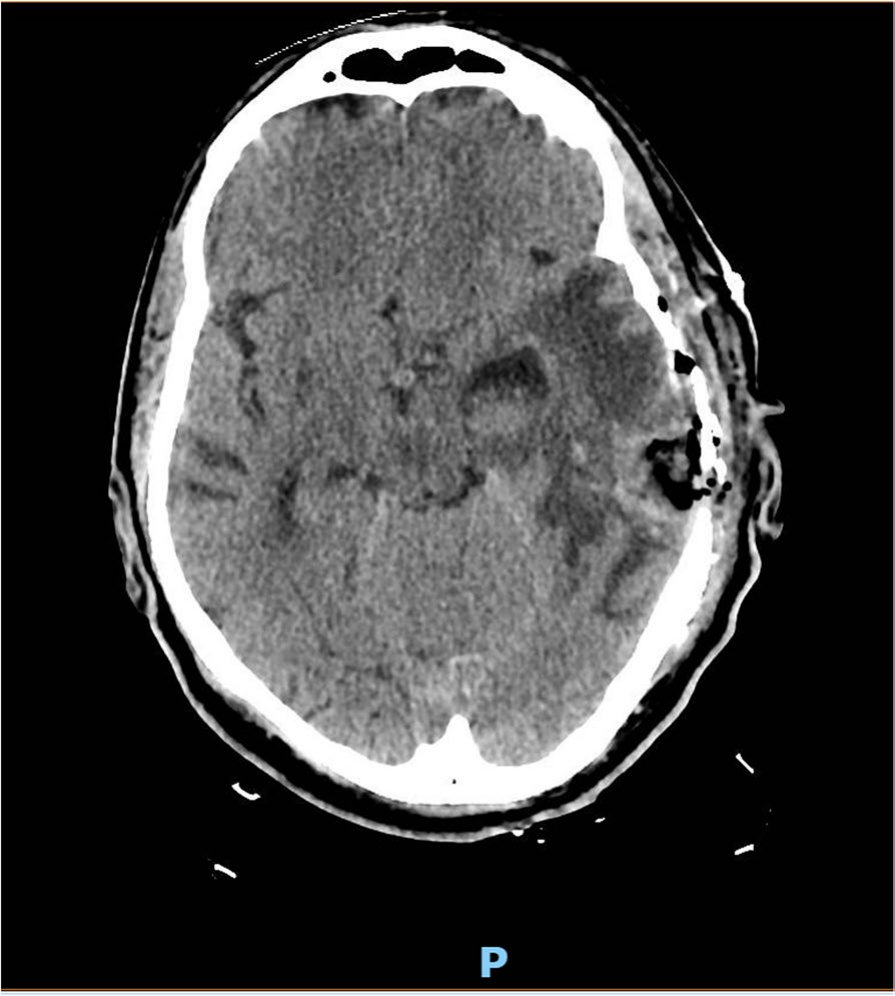
Brain CT-scan, showing left temporomandibular abscesses of 3.9 × 1.8 cm (lateral) and 2.4 × 1.5 cm (medial) respectively with hyperdense margins on baseline scans and post-contrast enhancement

A 55-year-old man with a history of alcohol abuse and recurrent otitis was found on the ground and brought to the emergency room with confusion and seizures. On physical examination, he presented with hypotension and severe hypothermia. He had a Glasgow Coma Scale (GCS) of 8 and was intubated for airway protection. The initial laboratory analysis revealed an increase in inflammatory markers (white blood cell [WBC] count 22.570 /μL, C-reactive protein [CRP] 218 mg/L [reference range 0–5], procalcitonin [PCT] 8.16 ng/mL) and blood tests were compatible with signs of rhabdomyolysis (creatin kinase [CK] 1602 UI/L, creatinine 2.35 mg/dl, lactate dehydrogenase [LDH] 376 U/L, myoglobin 3075 ng/ml). Brain computed tomography (CT) was performed, which showed two brain lesions in the left temporal-occipital site, measuring 3.9 × 1.8 cm and 2.4 × 1.5 cm respectively, with vasogenic edema and 0.9 cm left-to-right midline shift. Signs of inflammation of the paranasal sinuses were also reported (Fig. 1).

Chest and abdominal CT scan were also performed in order to rule out local pathologies and possible septic embolisms. Blood cultures resulted negative and transthoracic echocardiogram showed no vegetations or signs of endocarditis. Serology for HIV and *Toxoplasma gondii* resulted negative. Antiedema (mannitol) and anticonvulsant (valproate) therapy was initiated along with empiric antibiotic treatment with ceftriaxone, 2 g every 12 hours, metronidazole, 500 mg every 6 hours, and linezolid 600 mg every 12 hours. The culture of the brain abscess aspirate, collected during neurosurgery, identified *Actinomyces turicensis* and *Proteus mirabilis* on two different samples. Specifically, an intraoperative sample was collected in Amies elution medium and cultivated on three agar plates (*Sabouraud dextrose agar, Columbia CNA agar and MacConkey agar*), while another sample was collected in the absence of medium and cultivated on the same plates plus two additional ones (*Chocolate agar and microaerophilic Columbia CNA agar*). The plates were incubated at 37° degrees and first bacterial growth was observed at 36 hours. Microbiological identification was performed by MALDI-TOF (Bruker Biotyper®), showing high log (score) value (2.17 and 1.97 for each sample respectively). The antimicrobial susceptibility testing was performed by microdilution and Vitek-2 (bioMerieux®) automated system respectively for the anaerobic and the aerobic bacteria (Table 1).

**Table 1:** Antimicrobial susceptibility testing for *A. turicensis* and *P. mirabilis* isolated on patient

*A. turicensis*	Antibiotic	MIC	Susceptibility
	ampicillin	< 0.25 μg/mL	S
	ceftaroline	< 0.25 μg/mL	S
	linezolid	2 μg/mL	S
	moxifloxacin	< 0.125 μg/mL	S
	gentamicin	> 4 μg/mL	R
*P. mirabilis*			
	amoxicillin/clavulanic acid	8 μg/mL	S
	ceftazidime	< 0.12 μg/mL	S
	piperacillin/tazobactam	< 4 μg/mL	S
	meropenem	< 0.25 μg/mL	S
	gentamicin	< 1 μg/mL	S
	colistin	> 16 μg/mL	R
	ciprofloxacin	2 μg/mL	R

*MIC* Minimum inhibitory concentration *S* sensitive, *R* resistant

After obtaining the antimicrobial susceptibility test results, antibiotic therapy was simplified to ceftriaxone 2 g every 12 hours. Metronidazole and linezolid were discontinued.

**Fig. 2:**
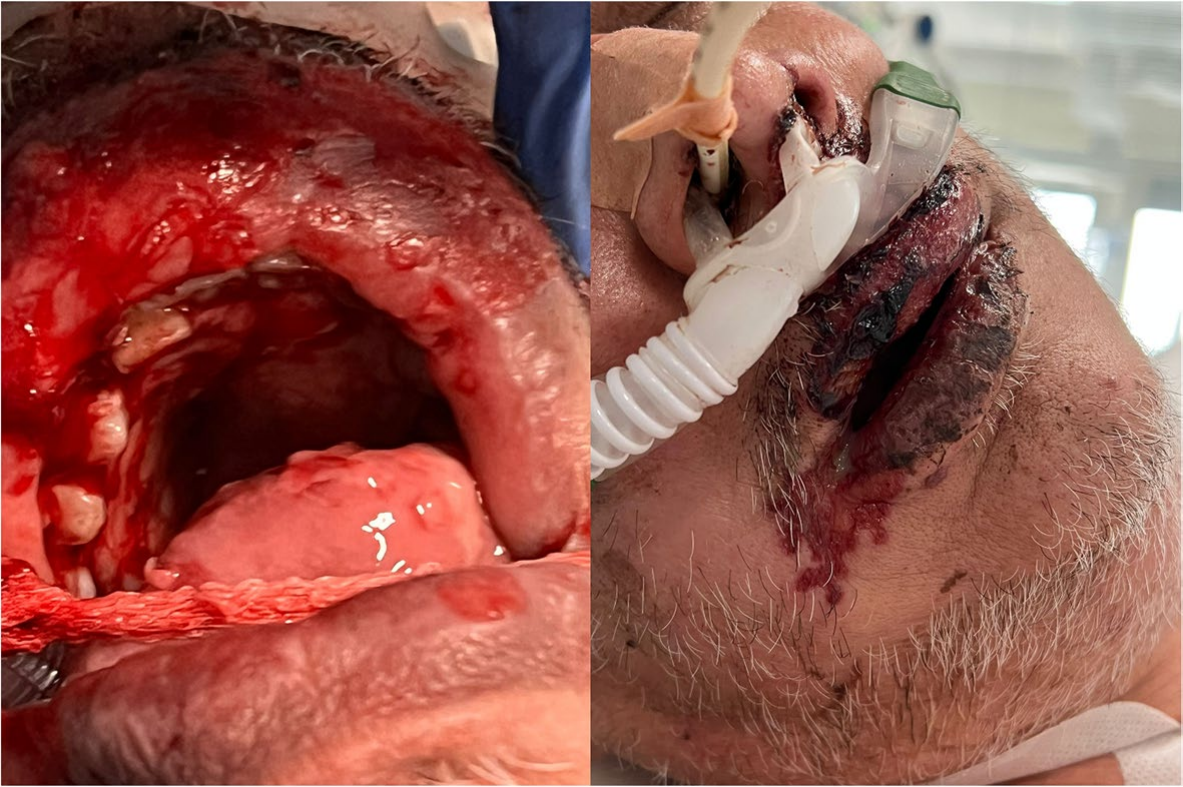
Vesiculopapular and necrotic lesions of the oral cavity and perioral area

After treatment optimization, the patient developed a fever and an initially vesiculopapular, then necrotizing, lesion of the upper lip and oral cavity (Fig. 2).

**Fig. 3:**
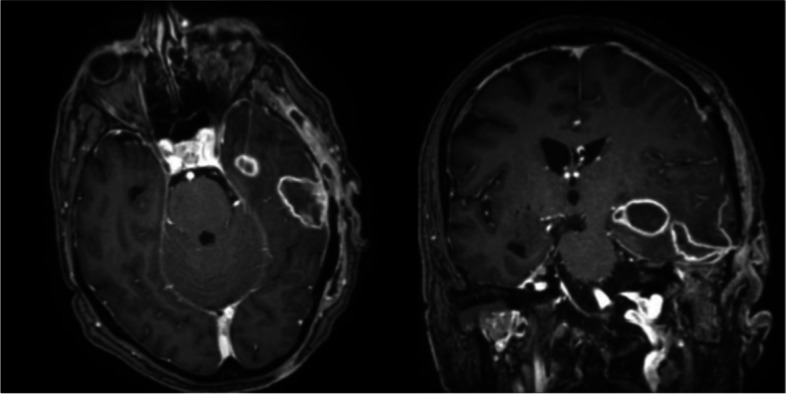
T1-weighted MR scans of brain, 5 weeks following neurosurgery

In the suspicion of a herpetic lesion, patient was started on acyclovir for 5 days, with progressive resolution of the lesion. To rule out a possible cutaneous involvement by *A. turicensis,* a wound swab was performed, resulting positive for *Herpes simplex virus*-1 (HSV-1) and a carbapenem-resistant *Acinetobacter baumannii*. Therefore, antimicrobial therapy was enhanced with the addition of ampicillin/sulbactam 3 g every 6 hours for improved coverage of both the brain abscess (*A. turicensis*) and the mucosal lesion isolate (*A. baumannii*). Five weeks after surgery, a brain magnetic resonance (MR) showed a reduction of the abscesses and resolution of edema and midline shift (Fig. 3).

The patient was then discharged to a neurorehabilitation facility with indication to continue the antimicrobial treatment with oral amoxicillin-clavulanate for a total of 8 weeks of therapy.

## Systematic review

### Materials and methods

The present study was conducted and reported according to the Preferred Reporting Items for Systematic Reviews and Meta-Analyses (PRISMA) statement [5].

### Search strategy and database selection

The search was conducted on the databases MEDLINE, EMBASE, Web of Science, CINAHL, Clinicaltrials.gov and Canadian Agency for Drugs and Technology in Health (CADTH), including all available records from inception to August 30th, 2023. Each included database was searched with the search term “*Actinomyces turicensis*” as an *all-terms* strategy. No filter was applied to the search engines. The search strategy as elaborated by the search engine, together with the corresponding records found divided by database is available in additional files (see Additional file 1).

Obtained records were merged on the online tool Rayyan, where duplicates were identified and removed from the included list. The first round of selection for relevance and eligibility was performed on the same platform [6]. Search and selection were performed in blind. Discrepancies in selection were resolved by discussion. A list of records obtained after the primary screening by title and abstract was then downloaded and entered into a computerized database for further analysis by reading the full text of the study. A final list of included records was then generated, and each study was examined for relevant data. Extracted information included author and journal information, year, study design, demographic information about included patient/s, site of infection, clinical presentation, diagnostic procedures, treatment, and outcome. Additional anamnestic information about possible predisposing conditions was also gathered. All extracted information was then summarized in figures and tables and added to the present study.

### Inclusion and exclusion criteria

Records were identified as eligible if they reported clinical data about infections by *A. turicensis*. No restrictions were made in terms of study design, peer-review, year of publication, country, language, patient age, or type of patient. In vitro or animal studies were excluded. Records reporting aggregated data only were excluded as well.

### Quality appraisal of included studies

Included studies were evaluated for their risk of bias by means of the most appropriate eligible reference scale when their design was either interventional or observational. For observational and randomized studies, the Newcastle-Ottawa scale (NOS) and the Cochrane Risk of Bias Tool 2 (ROB2) were used, respectively [7, 8]. The risk of bias analysis was performed, in blind, by AI, LVR and ADL. Discrepancies were solved by discussion.

## Results

**Fig. 4:**
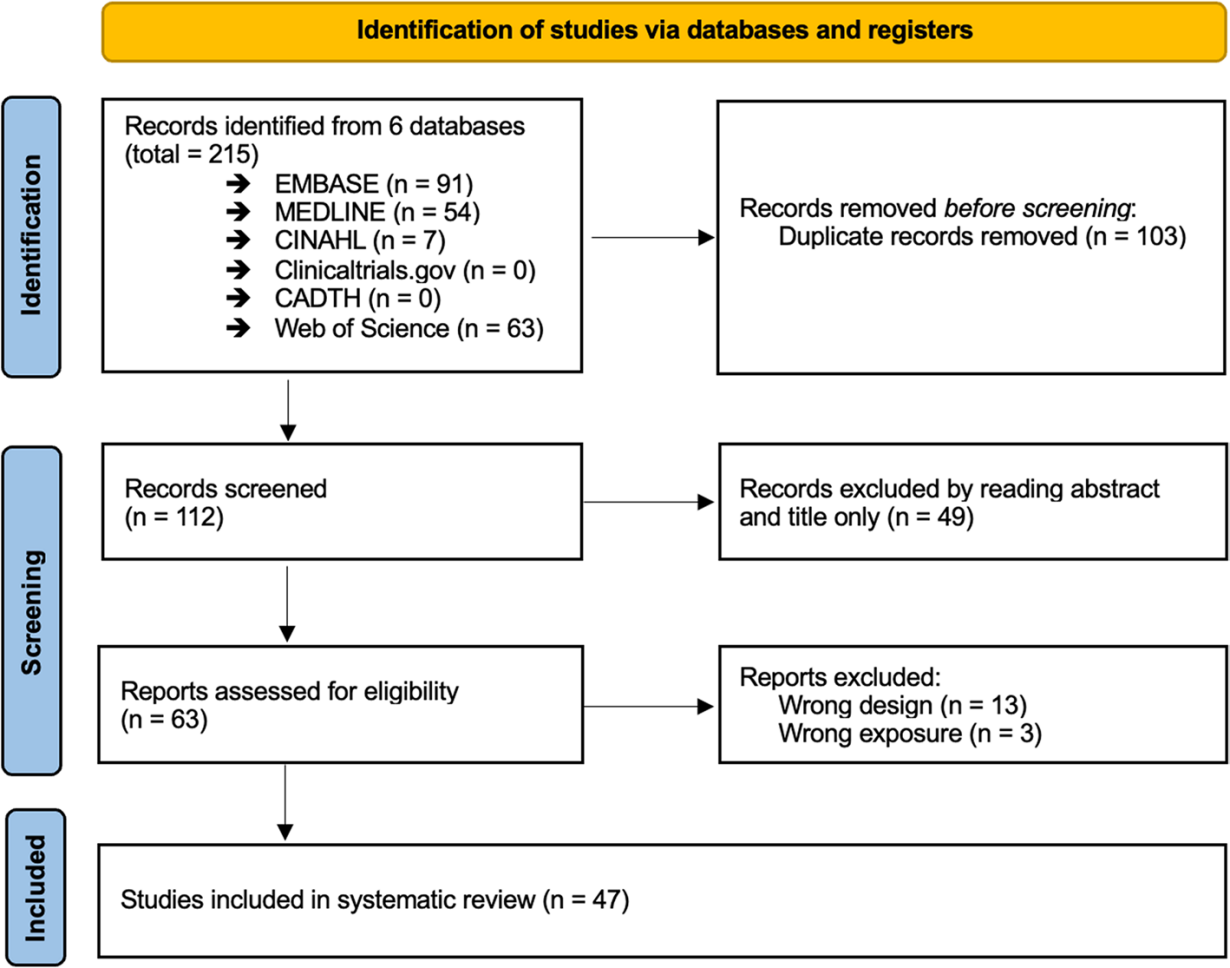
PRISMA flowchart of included studies

Our search on the six databases has identified 215 records, of which 103 were duplicates and were removed. Therefore, 112 records were screened for relevance and eligibility from the analysis of abstract and title only, resulting in 63 records. A subsequent examination of the relevant data in the full text was conducted, resulting in the exclusion of 16 records. At the end of the study selection process, 47 records were included in the systematic review. A flowchart describing the selection process is reported below (Fig. 4).

Included records were published between 2002 and 2023, with a prevalence in the last 5 years (26/47, 55%). Most of the studies were conducted in the USA (19/47, 40%), Europe (15/47, 32%) and China (3/47, 6%). Among the included records, we observed 43 Case reports, [1, 9–50] and 4 Case series [51–54], resulting in an overall population of 67 patients.

Clinical, demographics and microbiological records of the overall population are reported below (Tables 2 and 3).

**Table 2:** Demographic features and underlying conditions of the patients

References	Age	Sex	Predisposing risk factors	Immune system impairment
Panwar K et al., 2019 [9]	45	M	None	Diabetes, obesity
Saca J et al., 2023 [10]	49	M	Diabetic foot ulcer	Diabetes
Unigarro et al., 2023 [11]	58	F	Cervical cancer	Chemotherapy
Baher H et al., 2022 [12]	36	M	Endovenous drug use	None
Gandhi K et al., 2022 [13]	10	F	Surgical infection	None
Böttger S et al., 2022 [54]	74	M	Impacted decayed tooth with periodontitis	None
Fisher M et al., 2022 [14]	74	F	Pulmonary sequestration and COPD	None
Lin J et al., 2021 [15]	36	M	None	None
Sarumathi D et al., 2020 [55]	42	M	Nephrotic syndrome	Corticosteroids
Herrmann, AA et al., 2019 [17]	71	M	Stage IV esophageal cancer	Chemotherapy, immunotherapy
Lowry D et al., 2019 [18]	56	M	None	Diabetes, psoriatic arthritis on adalimumab treatment
Denham, J.D. et al., 2018 [19]	71	F	None	None
Snead, J.A. et al. 2018 [20]	79	M	Infected sacral decubitus ulcer	Prostate cancer
Gibson AL et al., 2018 [21]	3	F	Neurosurgical and spinal interventions	None
Elborno, D. et al., 2016 [22]	13	F	Microperforate hymen	None
Matela, A.et al., 2015 [23]	52	M	Dental procedure	None
Nickoloff, S et al., 2014 [56]	62	M	Poor dentition	Smoking
Shkolnik, I.et al., 2014 [25]	37	F	Poor dentition	Smoking, alcohol abuse
Palacios D et al., 2023 [26]	42	F	None	None
Doldán L et al., 2023 [27]	59	F	Cervical cancer	None
Cronin JT et al., 2023 [28]	70	M	Mini-open rotator cuff repair	Corticosteroid local injection
Tan CY et al., 2022 [51]	15 (IQR 8–52)	M (7), F (8)	N.A.	N.A.
Khan A et al., 2022 [29]	61	M	Benign prostatic hyperplasia	None
Mao TC et al., 2022 [30]	67	M	None	None
Tabaksert A et al., 2021 [1]	56	M	None	None
Nia A et al., 2021 [31]	42	M	None	None
Agrafiotis AC et al., 2021 [32]	51	M	None	Smoking, alcohol abuse, corticosteroids
Johnson SW et al., 2021 [33]	33	M	None	Obesity, diabetes
Barnes A et al., 2020 [34]	53	M	None	None
Jin W et al., 2020 [35]	50	F	None	None
Kansara T et al., 2020 [36]	52	F	None	None
Le Bihan A et al., 2019 [37]	43	F	Chronic lactation from breast	Smoking
Vassa N et al., 2019 [38]	61	M	None	Chemotherapy and radiation
Kocsis B et al., 2018 [39]	43	M	Mastoiditis	Alcohol abuse, smoking
Cobo F., 2018 [40]	44	F	Mastitis	None
Gatti M et al., 2017 [41]	64	F	None	Obesity
Eenhuis LL et al., 2016 [42]	42	F	Intra-uterine contraceptive device	None
Oh HB et al., 2015 [43]	25	F	None	None
Hagiya H et al., 2015 [44]	80	F	None	None
Kottam A et al., 2015 [45]	30	F	Intra-uterine contraceptive device	None
Miller S et al., 2014 [46]	5	M	Recurrent otitis media	None
Abdulrahman GO Jr. et al., 2015 [47]	22	F	Nipple piercing	Smoking
Ong C et al., 2012 [48]	73	F	None	Smoking
Chudácková E et al., 2010 [52]	28 (IQR 20–30)	M (4), F (3)	None	Diabetes (2), none (5)
Zautner AE et al., 2019 [49]	23	M	Femur hypoplasia	None
Attar KH et al., 2007 [53]	33	F	Bilateral nipple piercing	Steroid, smoke, obesity
Riegert-Johnson DL et al., 2002 [50]	59	M	Dental care	None

*COPD* chronic obstructive pulmonary disease, *IQR* interquartile range, *N. A* not available

**Table 3:** Clinical presentation and microbiological findings

References	Infection	Microbiological findings	Coinfections	Symptoms
Panwar K et al., 2019 [9]	Necrotizing fasciitis	Monomicrobial	Nil	Nausea, vomit, fever
Saca J et al., 2023 [10]	Osteomyelitis and necrotizing fasciitis	Polymicrobial	*S. agalactiae, P. denticola, S. moorei*	Foot pain, fever, tachycardia
Unigarro et al., 2023 [11]	Septic shock after uterine perforation	Monomicrobial	Nil	Dysuria, abdominal pain, nausea, vomit, drowsyness, hypotension
Baher H et al., 2022 [12]	Pleural empyema	Monomicrobial	Nil	Fever, tachycardia, tachypnea, hypotension
Gandhi K et al., 2022 [13]	Abscess of the cartilagineous helix	Monomicrobial	Nil	Pain, erythema at previous surgical site
Böttger S et al., 2022 [54]	Odontogenic craniofacial necrotizing fasciitis	Polymicrobial	*B. thetaiotaomicron, S. epidermidis*	Black blisters, anesthesia of the skin, livid erythema
Fisher M et al., 2022 [14]	Pulmonary abscess	Monomicrobial	Nil	Dyspnea and cough
Lin J et al., 2021 [15]	Abscess of the buttocks	Monomicrobial	Nil	Pain, erythema, purulent cutaneous discharge
Sarumathi D et al., 2020 [55]	UTI	Monomicrobial	Nil	Fever, dysuria, and loose stools
Herrmann, AA et al., 2019 [17]	Spinal epidural abscess	Polymicrobial	*E. cloacae, S. milleri*	Back pain, fever
Lowry D et al., 2019 [18]	Pulmonary abscess	Monomicrobial	Nil	Dyspnea
Denham, J.D. et al., 2018 [19]	Pyometra	Monomicrobial	Nil	Purulent vaginal discharge
Snead, J.A. et al. 2018 [20]	Bacteremia	Monomicrobial	Nil	Fever, chills, tachycardia, hypotension, altered mental status
Gibson AL et al., 2018 [21]	Epidural abscess	Polymicrobial	*A. europaeus*	Fever, lethargy
Elborno, D. et al., 2016 [22]	Tubo-ovarian abscess	Monomicrobial	Nil	N.A.
Matela, A.et al., 2015 [23]	Pulmonary abscess	Polymicrobial	*S. viridans*	Chest pain, fever
Nickoloff, S et al., 2014 [56]	Empyema	Monomicrobial	Nil	Chest pain, fever, weight loss
Shkolnik, I.et al., 2014 [25]	Pulmonary abscess	Monomicrobial	Nil	Weight loss, cough, chest pain
Palacios D et al., 2023 [26]	Recurrent peri-clitoral abscess	Monomicrobial	Nil	Recurrent peri-clitoral mass
Doldán L et al., 2023 [27]	Para-uterine abscess	Monomicrobial	Nil	Purulent vaginal discharge, fever
Cronin JT et al., 2023 [28]	Surgical site infection	Monomicrobial	Nil	Purulent surgical wound dehiscence
Tan CY et al., 2022 [51]	Pilonidal (11), Perianal (4)	Monomicrobial (1), polimicrobial (14)	Mixed anaerobes*, S. milleri, S. aureus, Citrobacter* spp.*, Coliform*	N.A.
Khan A et al., 2022 [29]	Fournier’s gangrene	Polymicrobial	*H. haemolyticus, S. anginosus, P harei*	Diarrhea, fever, penile swelling, dysuria, hematuria, hypotension
Mao TC et al., 2022 [30]	Fournier’s gangrene	Monomicrobial	Nil	Scrotum swelling
Tabaksert A et al., 2021 [1]	Parapharingeal and mediastinal abscess	Polymicrobial	*E. faecalis, S. anginosus, S. constellatus*	Fever, dysfagia
Nia A et al., 2021 [31]	Hip abscess	Polymicrobial	*F. nucleatum*	Pain, fever
Agrafiotis AC et al., 2021 [32]	Pleural empyema	Polymicrobial	*F. necrogenes, M. micros*	N.A.
Johnson SW et al., 2021 [33]	Pleural empyema	Polymicrobial	*F. nucleatum*	Chest pain, cough, fever
Barnes A et al., 2020 [34]	Prostatic abscess and Mandibular abscess	Polymicrobial	*Peptostreptococcus* spp.	Shock, inguinal pain, fever, vomit, dysuria
Jin W et al., 2020 [35]	Adrenal gland abscess	Polymicrobial	*E. coli, P. mirabilis*, plus others in mNGs	Back pain, fever
Kansara T et al., 2020 [36]	Pyelonephritis and abscess	Monomicrobial	Nil	Abdominal pain, vomit, fever
Le Bihan A et al., 2019 [37]	Breast abscess	Polymicrobial	*P. harei*	Breast swelling
Vassa N et al., 2019 [38]	Ludwig angina	Monomicrobial	Nil	Oral bleeding
Kocsis B et al., 2018 [39]	Meningitis	Monomicrobial	Nil	Unconsciousness, fever
Cobo F., 2018 [40]	Breast abscess	Monomicrobial	Nil	Pain, fever
Gatti M et al., 2017 [41]	Abdominal wall	Monomicrobial	Nil	Hypotension, necrotic abdominal wall
Eenhuis LL et al., 2016 [42]	Peritonitis	Monomicrobial	Nil	Hypotension, fever, abdominal pain
Oh HB et al., 2015 [43]	Pilonidal abscess	Polymicrobial	*P. bivia, Peptostreptococcus* spp.	Swelling of sacral region, fever
Hagiya H et al., 2015 [44]	Pyometra	Polymicrobial	*C. clodtridioforme*	Fever
Kottam A et al., 2015 [45]	Endocarditidis and pelvis and liver microabscesses	Monomicrobial	Nil	N.A.
Miller S et al., 2014 [46]	Cerebellar abscess	Polymicrobial	*P. mirabilis, P. harei, B. thetaiotaomicron, A. hydrogenalis*	Otorrhoea, anorexia, vomit, lethargy
Abdulrahman GO Jr. et al., 2015 [47]	Breast abscess	Polymicrobial	*P.harei*	Breast pain
Ong C et al., 2012 [48]	Left iliac fossa and liver abscesses	Monomicrobial	Nil	Abdominal pain, fever
Chudácková E et al., 2010 [52]	Pilonidal (2), cutaneous (2), anal (1), perianal (1), gas gangrene (1)	Monomicrobial (2), polimicrobial (5)	*B. ureolyticus, F. nucleatum, S. milleri, P. anaerobius, S. aureus, P. acnes, Prevotella* spp.	N.A.
Zautner AE et al., 2019 [49]	Fistula of the knee	Polymicrobial	*A. europaeus*	Swelling of the knee
Attar KH et al., 2007 [53]	Breast abscess	Monomicrobial	Nil	Pain, sweeling, fever
Riegert-Johnson DL et al., 2002 [50]	Hepatic abscess	Polymicrobial	*B. fragilis*	Fever, vomit

Key: *mNGs* metagenomic next-generation sequencing, *N.A.* not available, *UTI* urinary tract infection

Some of the included cases did not provide enough information about immunosuppression conditions, symptoms, or treatments; therefore, the lack of data was considered when calculating the incidences, to minimize underestimation of the data.

### Demographic features and underlying conditions

Published cases showed an almost equal distribution of males and females (35 vs. 32) with a median age of 42 (IQR 23–57). From the analysis of the patient anamnestic data, 21 patients (21 out of the 52 patients for which data was available, i.e. 40%) resulted to have had some cause of comorbidity or immunosuppression, particularly smoking (9), diabetes (6), obesity (5), chemotherapy or immunotherapy (4), high dose steroids (3), alcohol abuse (3). Moreover, in relation to the site of infection, a supposed predisposing condition was reported in 27 patients (27/52, 52%). No information about predisposing condition or immunosuppression were reported for 15 patients.

### Site of infection and associated symptoms

Among the overall population, we observed 21 infections of the anogenital district, 12 Acute Bacterial Skin and Skin Structure Infections (ABSSSI) of which 2 were defined as Fournier’s gangrene, 8 lung infections (4 empyema and 4 abscesses), 6 gynecological infections, 5 infections of the cervicofacial district, 4 infections of the breast, 4 abdominal infections (1 peritonitis, 2 liver abscesses, 1 infection of the adrenal gland), 3 urinary tract infections, 2 infections of the vertebral column, 2 central nervous system infections, 1 endocarditis. One patient had both the cervicofacial region and urinary tract infections. Interestingly, 36 (36/67, 54%) infections presented as abscesses and 7 infections (7/67, 19.4%) presented with a concomitant bacteremia. Among the symptoms described at admission, fever (25 out of the 42 patients for which such data were available, i.e. 60%), local pain (18/42, 43%), local swelling and erythema (8/42, 19%), vomiting (6/42, 1%), dysuria (4/42, 10%), were the most frequently reported. Furthermore, 7 patients (7/42, 17%) presented with hypotension or shock and 5 patients (5/42, 12%) presented with altered state of consciousness. In the case of 25 patients, no information about symptoms was reported.

### Microbiology

In all cases where the data were available, the microbiological identification of *A. turicensis* was allowed by culture examination. This was conducted on tissue samples (31/62, 50%), purulent drainage fluid (14/62, 22.5%), intraoperative samples (6/62, 9,6%), blood samples (7/62, 11.2%), Broncho-Alveolar Lavage (BAL) fluid (2/62, 3.2%), cerebrospinal fluid (1/62, 1.6%), urine sample (1/62, 1.6%). Fifty-seven percent of the infections were polymicrobial (*n* = 38). Reported co-infections were identified by tissue/pus culture or molecular assays and are reported in Table 3. Co-infecting agents were almost invariably part of the anaerobic flora.

### Treatment

Out of the 67 cases described in the literature, abscess drainage was performed in 10 patients (15%), surgical debridement was performed in 41 cases (61%), an antibiotic approach without surgery was chosen for 15 patients (22%), while no information about surgical procedures was reported for one patient. Surgery was considered curative, i.e. without any antibiotic therapy, in 8 out of 67 patients, though insufficient data was reported for the antibiotic treatment for 11 patients. Specifically, 4 received an unspecified broad-spectrum antibiotic regimen, while for 7 patients no data was reported.

In the other 48 cases, a wide range of antibiotic use was reported, as summarized in Table 4.

**Table 4:** Treatment strategies and clinical outcome

References	Source control	Administered antibiotics	Duration of therapy (days)	Outcome
Panwar K et al., 2019 [9]	Surgical debridement	VAN, TZP	N.A.	Full recovery
Saca J et al., 2023 [10]	Surgical debridement,	AMC, SAM	N.A.	Recurrence and superinfection
Unigarro et al., 2023 [11]	None	CARBA, LZD, CLI	9	Full recovery
Baher H et al., 2022 [12]	None	AMC, MTZ	N.A.	Full recovery
Gandhi K et al., 2022 [13]	None	AMC	180	Full recovery
Böttger S et al., 2022 [54]	Surgical debridement	CARBA	N.A.	Full recovery
Fisher M et al., 2022 [14]	None	N.A.	N.A.	Full recovery
Lin J et al., 2021 [15]	None	STX	90	Full recovery
Sarumathi D et al., 2020 [55]	None	MTZ, AMP	N.A.	Full recovery
Herrmann, AA et al., 2019 [17]	None	N.A.	N.A.	Death
Lowry D et al., 2019 [18]	None	N.A.	N.A.	Full recovery
Denham, J.D. et al., 2018 [19]	None	AMC	180	Full recovery
Snead, J.A. et al. 2018 [20]	None	TZP	42	Full recovery
Gibson AL et al., 2018 [21]	N.A.	N.A.	N.A.	N.A.
Elborno, D. et al., 2016 [22]	Drainage	AMX, MTZ	365	Full recovery
Matela, A.et al., 2015 [23]	Surgical debridement	TZP, AMC	N.A.	Full recovery
Nickoloff, S et al., 2014 [56]	Drainage	AMC	N.A.	Full recovery
Shkolnik, I.et al., 2014 [25]	Drainage	CRO, MTZ	42	N.A.
Palacios D et al., 2023 [26]	Drainage	AMX	14	Recurrence
Doldán L et al., 2023 [27]	Drainage	AMX	90	Full recovery
Cronin JT et al., 2023 [28]	Surgical debridement	AMX	420	Full recovery
Tan CY et al., 2022 [51]	Surgical debridement	N.A.	0 (0–6.5)	N.A.
Khan A et al., 2022 [29]	Surgical debridement	TZP, VAN, CLI, SAM, AMC	21	Full recovery
Mao TC et al., 2022 [30]	Surgical debridement	CFP, TZP, CLI	N.A.	Full recovery
Tabaksert A et al., 2021 [1]	Surgical debridement	CARBA, MTZ, AMX	180	Full recovery
Nia A et al., 2021 [31]	Surgical debridement	AMC, MTZ	42	Full recovery
Agrafiotis AC et al., 2021 [32]	Surgical debridement	AMC	180	Full recovery
Johnson SW et al., 2021 [33]	Dreinage	SAM, AMC	180	Full recovery
Barnes A et al., 2020 [34]	Surgical debridement	VAN, TZP, SAM, CRO, AMC	210	Full recovery
Jin W et al., 2020 [35]	Drainage	CARBA	91	Full recovery
Kansara T et al., 2020 [36]	None	MTZ, CARBA, VAN, CRO	15	Full recovery
Le Bihan A et al., 2019 [37]	None	AMX, MTZ	70	Full recovery
Vassa N et al., 2019 [38]	None	VAN, TZP, PEN, LVX, MTZ, SAM	42	Full recovery
Kocsis B et al., 2018 [39]	Surgical debridement	CRO, VAN, AMP	N.A.	Death
Cobo F., 2018 [40]	None	AMX	10	Full recovery
Gatti M et al., 2017 [41]	Surgical debridement	DAP, RIF, TZP, AMP	35	Full recovery
Eenhuis LL et al., 2016 [42]	Surgical debridement	CRO, GEN, and MTZ, PEN,	210	Full recovery
Oh HB et al., 2015 [43]	Surgical debridement	AMC	7	Full recovery
Hagiya H et al., 2015 [44]	Drainage	SAM	30	Full recovery
Kottam A et al., 2015 [45]	Surgical debridement	PEN, CRO, MTZ, CARBA	60	Full recovery
Miller S et al., 2014 [46]	Surgical debridement	CTX, MTZ, PEN,CIP, AMX	210	Full recovery
Abdulrahman GO Jr. et al., 2015 [47]	Drainage	AMC, PEN, AMX	194	Full recovery
Ong C et al., 2012 [48]	None	PEN, AMX	180	Full recovery
Chudácková E et al., 2010 [52]	Surgical debridement	N.A.	N.A.	N.A.
Zautner AE et al., 2019 [49]	Surgical debridement	PEN, GEN	14	Recurrence and superinfection
Attar KH et al., 2007 [53]	Surgical debridement	VAN, CXM	21	Full recovery
Riegert-Johnson DL et al., 2002 [50]	Drainage	CRO, MTZ	150	Full recovery

*VAN* vancomycin, *TZP* piperacilline/tazobactam, *SAM* ampicillin/sulbactam, *AMC* amoxicillin/clavulanic acid, *CARBA* carbapenem, *LZD* linezolid, *CLI* clindamycin, *MTZ* metronidazole, *STX* trimethoprim/sulfamethoxazole, *AMP* ampicillin, *AMX* amoxicillin, *CRO* ceftriaxone, *CFP* cefoperazone, *PEN* penicillin, *LVX* levofloxacin, *DAP* daptomycin, *RIF* rifampin, *GEN* gentamicin, *CTX* cefotaxime, *CIP* ciprofloxacin, *CXM* cefuroxime

Broad-spectrum antibiotics, active on both Gram-positive and Gram-negative bacteria, were the most frequent first choice treatment, favoring intravenous administration in severe infections. Particularly, piperacillin/tazobactam was used in 7 patients, vancomycin was prescribed in 6 cases, carbapenems where the treatment of choice in 5 patients, while metronidazole or cephalosporin were used in 3 cases each. Regarding targeted therapy, the most frequently administered antibiotics were amoxicillin/clavulanate (n.17 cases), amoxicillin (n.13 cases), ampicillin/sulbactam (n.6 cases), penicillin (n. 6 cases) and ampicillin (n. 4 cases). Metronidazole (n.15 cases) or cephalosporin (n.6 cases) were added in case of suspected or documented polymicrobial infections.

Regarding the overall duration of therapy, data were available for 46 out of 67 patients. Mean treatment duration was 80 days, while median duration was 38.5 days (IQR 7.5–172.5). Shorter treatment, i.e. less than 1 month, was the most frequently observed (14/46, 30%), followed by a duration of 1–3 months (10/46, 22%), 3–6 months (8/46, 17%) and more than 6 months (6/46, 13%). The remaining cases underwent no antimicrobial therapy as surgery was considered curative (8/46, 17%). As expected, longer treatments were reported in cases of abscesses.

### Outcome

Among the included studies, clinical outcome data were available for 44 out of 67 cases (65.6%). Thirty-nine patients (89%) showed a full recovery, while 3 patients (7%) experienced recurrence or superinfection and 2 patients (5%) died.

## Discussion and conclusions

To the best of our knowledge, this is the first case in the literature of a brain abscess caused by *A. turicensis* and *P. mirabilis* in an adult patient. Brain involvement in actinomycosis is uncommon [57, 58], generally resulting from hematogenous spread or contiguous infection of the ear, sinus, and cervicofacial region [46, 58, 59]. In our case, the brain CT showed inflammation of the paranal sinuses but excluded ear involvement, even if a history of frequent otitis was reported.

Brain abscesses caused by opportunistic pathogens are frequently in patient with Human Immunodeficiency virus (HIV) infection or other causes of immunosuppression, whereas bacteria are the most common cause in immunocompetent patients [60]. While actinomycosis is a non-opportunistic disease, central nervous system involvement is very rare. Therefore, possible causes of immunosuppression must always be excluded. Our patient had a history of alcohol abuse [61, 62], which is considered a pro-inflammatory and nutritionally impaired condition often associated with immune deficiency.

The diagnosis of actinomycosis is challenging and requires an invasive approach for diagnosis. Literature suggests a surgical intervention for any brain abscess measuring at least 2.5 cm in diameter [63, 64]. Our patient underwent surgical excision of abscesses with consequent microbiological identification. Brain abscesses are frequently polymicrobial [46, 65, 66]; indeed *P. mirabilis* was also identified in our case [66].

Furthermore, growth of *Actinomyces* is generally slow and the bacteriological identification is difficult. Culture could frequently result sterile due to previous antibiotic therapy, concomitant microorganisms and inadequate sampling or incubation conditions. Surgical sampling of biopsy or pus seems to be the most appropriate clinical specimen [3].

Although often difficult to diagnose, actinomycosis is generally readily treatable, showing susceptibility to many antimicrobials including β-lactams, clarithromycin, erythromycin, doxycycline, and clindamycin. Therefore, thanks to the wide susceptibility and availability of treatment, several are the drugs of choice and there is no univocal indication. However, penicillin G or amoxicillin are the most used [3].

In our case, ceftriaxone was considered as target therapy with addiction to ampicillin/sulbactam for a week, as strengthening of the brain abscesses treatment. The prompt clinical and laboratory response in our patient allowed the switch to oral therapy with amoxicillin-clavulanic acid, which has proven to be non-inferior to standard intravenous treatment [67].

Our systematic review of the literature identified 47 articles reporting infections caused by *A. turicensis.* All included records are case reports (43) and case series (4), with an increased number of published papers in the last 20 years, probably due to the improvement of microbiological techniques, spectrometry, and molecular assay, that allow to better identification of *Actinomyces* species. Since the diagnosis of actinomycosis requires bacteriological identification, a lack of correct microbiological data, in the past, may have led to a misinterpretation of the risk and an underestimate of the incidence.

Although *A. israelii* is the main cause of disease within the species [4], we identified 67 cases of infections due to *A. turicensis*. From the present literature revision, most *A. turicensis* cases were anogenital, gynecological and urinary tract infections (30), lung infections (8) or cervicofacial infections (5).

As reported in the literature, actinomycosis is generally due to local dissemination of the pathogen rather than hematogenous spread [4]. Among the analyzed articles, a concomitant bacteremia was indeed found in 10% (7/67) of cases only, while a predisposing condition of local dissemination was supposed in at least 40% (27/52) of cases. Notably, while actinomycosis is a non-opportunistic disease, a reason for immune system impairment has been found in at least 52% (21/52%) of the cases.

Interestingly, only two central nervous system infections were reported among the included records, both presenting a history of ear infections (i.e. mastoiditis and otitis). In our cases, although a previous history of recurrent otitis was reported, no acute ear infection was present at patient admission. Concerning treatment options and outcome, a wide range of therapies is reported and a relatively low mortality (5%), confirming to be a readily treatable infection when promptly diagnosed [2].

In 76% of cases drainage or surgical debridement was performed, representing not only a therapeutical approach but also as a diagnostic procedure.

In conclusion, diagnosis of actinomycosis is challenging and requires prompt microbiological identification. Surgical excision or drainage together with long-term antibiotics is essential to achieve clinical recovery. Further investigations are needed to assess the optimal antibiotic regimen and its duration.

### Supplementary Information

**Additional file 1.**

## Data Availability

All data generated or analyzed during this study are included in this article and its supplementary materials.

## Abbreviations

ABSSSI: Acute bacterial skin and skin structure infection
AMC: Amoxicillin/clavulanic acid
AMP: Ampicillin
AMX: Amoxicillin
BAL: Broncho alveolar lavage
CADTH: Canadian agency for drugs and technology in health
CARBA: Carbapenem
CFP: Cefoperazone
CIP: Ciprofloxacin
CK: Creatine Kinase
CLI: Clindamycin
CRO: Ceftriaxone
CRP: C Reactive Protein
CT: Computed tomography
CTX: Cefotaxime
CXM: Cefuroxime
DAP: Daptomycin
GEN: Gentamicin
HIV: Human immunodeficiency virus
HSV: Herpes Simplex Virus
IQR: InterQuartile Range
LDH: Lactate dehydrogenase
LVX: Levofloxacin
LZD: Linezolid
MALDI-TOF: Matrix assisted laser desorption ionization – time of flight
MIC: Minimum inhibitory concentration
MR: Magnetic resonance
MTZ: Metronidazole
N.A.: Not Available
NOS: Newcastle-ottawa scale
GCS: Glasgow coma scale
PCT: Procalcitonin
PEN: Penicillin
PRISMA: Preferred reporting items for systematic reviews and meta-analyses
RIF: Rifampin
S: Sentitive
SAM: Ampicillin/sulbactam
STX: Trimethoprim/sulfamethoxazole
R: Resistant
TZP: piperacilline/tazobactam
VAN: Vancomycin
