# Supplementary material for: Brain abscess caused by Actinomyces turicensis in a non-immunocompromised adult patient: a case report and systematic review of the literature
Source: BMC Infect Dis. 2024 Jan 20;24:109. doi: 10.1186/s12879-024-08995-w (PMC10799506; doi:10.1186/s12879-024-08995-w)
Supplement: Supplementary file 1 — Additional file 1. [file 12879_2024_8995_MOESM1_ESM.docx]

Embase, search strategy:

'Actinomyces turicensis'/exp OR 'actinomyces turicensis'

Search performed on Aug 30th, 2023. 91 records. No filter was applied on the search engine.

Pubmed, search strategy:

“Actinomyces turicensis”

Search performed on Aug 30th, 2023. 54 records. No filter was applied on the search engine.

ClinicalTrials.gov, search strategy:

“Actinomyces turicensis”

Search performed on Aug 30th, 2023. 0 records. No filter was applied on the search engine.

CINAHL search strategy:

“Actinomyces turicensis”

Search performed on Aug 30th, 2023. 7 records. No filter was applied on the search engine.

CADTH search strategy:

“Actinomyces turicensis”

Search performed on Aug 30th, 2023. 0 records. No filter was applied on the search engine.

Web Of Science search strategy:

“Actinomyces turicensis”

Search performed on Aug 30th, 2023. 63 records. No filter was applied on the search engine.
